# Informing hard-to-reach immigrant groups about COVID-19—Reaching the Somali population in Oslo

**DOI:** 10.1093/jrs/feab053

**Published:** 2021-05-08

**Authors:** Jan-Paul Brekke

**Affiliations:** Institute for Social Research, Box 3233, Oslo 0208, Norway

**Keywords:** Covid, communication, immigrant groups, trust

## Introduction

The COVID-19 pandemic has highlighted the need for governments to be able to reach all groups of residents with potentially vital information. If some groups do not get access to, or do not understand, public advice on how to prevent infection, this may have far-reaching consequences for the society as a whole. The current pandemic underscores the need for a deeper understanding of the dynamics and effectiveness of health campaigns directed at hard-to-reach immigrant groups.

This article provides a case study of an information campaign directed at people of Somali decent living in the worst hit district of Oslo, the capital of Norway. The Somalis were the immigrant group most affected by the COVID-19 in Norway during the first wave of the pandemic. The campaign used selected Somali-speaking ‘ambassadors’ as well as videos and network methodology to reach those within the Somali population who are least integrated into Norwegian society. The lessons learned from this case may both inform the theory of information campaigns and provide practical lessons learned for other groups in later high-risk information-need situations.

At the height of the first wave of the COVID crisis in Norway, in early April 2020, the Norwegian Institute of Public Health (NIPH) published numbers showing that the Somali population in Oslo was overrepresented among those infected with COVID-19 (https://www.aftenposten.no/osloby/i/wPG8zo/raymond-johansen-mange-av-de-smittede-i-oslo-har-innvandrerbakgrunn (accessed 15 August 2020) (Indseth et al. 2020). On 1 April 2020, 4655 people were infected in Norway. Of these, 829 were immigrants. The top three nationalities were Somali (201), Swedish (45) and Pakistani (41). https://www.dagbladet.no/studio/siste-nytt-om-coronaviruset/606?post=30518 (accessed 15 August 2020). At the same time, reports from neighbouring Sweden found that by the end of March, one-third of COVID-related deaths were of people of Somali origin. The news elicited a hectic search among national and local government bodies and within the Somali community itself for effective measures, including for communication platforms and campaign designs.

The news of overrepresentation elicited a range of theories on why the virus hit the Somali group particularly hard. These included the group being exposed through front-line work, such as taxi drivers and nurses, congested living conditions and an intra-group care culture. In addition, calls came for tailored information campaigns.

The ambassador project was one of many simultaneous efforts during the spring of 2020 in Norway and in the city of Oslo to communicate anti-infection information to ethnic minority populations. National institutions, including the Norwegian Institute for Public Health, local governments, NGOs, ethnic community organizations and private persons initiated a range of measures. These included videos, translated documents, posters, face-to-face campaigning in apartment buildings, stores, and restaurants; and web-based campaigns on SMS and social media.

Health communication is a flourishing academic field including studies on the communication of health-related information during times of crisis (Gamhewage 2014; Kim and Kreps 2020), and on the relationship between media and medicine (Briggs and Hallin 2016). In this article, the Oslo Somali campaign will be discussed using theoretical concepts from communication and campaign theory (Macnamara 2014), including the distinction between input (senders, content, formats), output (platforms, products) and outcomes (changes of attitudes, beliefs, and behaviours); studies of trust (Rothstein 2000; Eek and Rothstein 2005) and intercultural communication (Watson 2008).

Research on public crisis communication strategies often points to the idea that in order to be effective, communication presupposes both the vertical trust between the people and the authorities and the horizontal trust between people (Eek and Rothstein 2005). One could argue that information regarding one’s own health and public health could be particularly dependent on trust (https://www.dagbladet.no/kultur/tillit-kan-stanse-smitten/72283180; https://www.aftenposten.no/norge/i/K3957e/statsminister-erna-solbergs-tale-til-folket-i-forbindelse-med-Covidkrisen). Research has found that communicative trust can be distilled down to the actor’s trustworthiness, that is, their ability, integrity and benevolence (Mayer *et al*. 1995).

A 2016 survey mapping levels of trust among immigrant populations in Norway found high levels of vertical, or institutional, trust among people of Somali descent (Wiggen 2016). This group’s level of horizontal trust, trust toward other people, was in line with that of other immigrant groups, while somewhat lower than that of the majority population (Blom 2017). Surveys among immigrant populations can be challenging due to factors such as language, difficulties in reaching recent arrivals and lack of trust in the surveying institution (Font and Mendez 2014). Mapping the attitudes of hard-to-reach segments of such populations, including individuals who not follow national or local news, will be even harder. Therefore, the Norwegian survey results on trust may not reflect levels of institutional and interpersonal trust within the hard-to-reach subgroup. This study raises a third dimension of trust relevant to the communication of anti-infection information, namely, intra-group trust, specifically, vertical and horizontal trust within the Somali community. These dimensions were not covered in the Norwegian survey.

Based on 10 qualitative interviews with key informants conducted at the height of the COVID crisis in Oslo (in April 2020), this article asks, what are the prerequisites for reaching hard-to-reach immigrant communities with information about infection prevention?

To answer this question, the article first describes the context for the case of the Somali community in this particular district in Oslo. Next, there is a presentation of relevant theoretical concepts, a description of the methodology and data and an explanation of the study’s results. The final discussion section focuses on learning points gleaned from the combination of the theoretical concepts and the empirical data.

## Background

### Immigration to Norway

Over the last 50 years, immigration to Norway has been a mix of labour immigration from Asia and later from Europe, and humanitarian and family immigration from Asia, Africa and Latin America (Kjedstadlie and Brochmann 2014). In 2020, 18% of the total population of 5.4 million were foreign-born or children of two foreign-born parents (https://www.ssb.no/innvandring-og-innvandrere/faktaside/innvandring).

### The Somali Population in Oslo

The population of Somali descent in Norway constituted the largest non-European immigrant group (43,000) in Norway in 2020. One in three of this population were Norwegian born with two Somali-born parents (https://www.ssb.no/innvandring-og-innvandrere/faktaside/innvandring). In Oslo, a city of 680,000 people, 16,000 were born in Somalia (https://www.oslo.kommune.no/statistikk/befolkning/landbakgrunn/#gref). In the city, one in four were of immigrant descent (first or second generation). The city district selected for this study was that with the largest Somali community in Oslo and the area with the highest density of persons with Somali descent in Norway. Six per cent in the district were either Somali immigrants or second generation (https://bydelsfakta.oslo.kommune.no/bydel/gamleoslo/innvandrerbefolkningen). The majority of the Somali immigrants in Norway arrived around 2000 as asylum seekers and later as family migrants. The relatively short time since arrival contributes to the group scoring low on integration indicators, such as being outside the labour market and with poor housing conditions (https://www.ssb.no/inntekt-og-forbruk/artikler-og-publikasjoner/nesten-111-000-barn-vokser-opp-med-vedvarende-lave-husholdningsinntekter). Together, these are non-integration factors, believed to be prominent in the hard-to-reach subgroups within the Somali community.

Research on the Norwegian-Somali community in Norway over the past 15 years has covered a range of topics. These include the group’s social traditions based on family lineages, and marked by mobility, equality and individualism (Assal 2006; Fuglerud and Engebrigtsen 2006: 1130); the group’s challenges with regard to employment, drop-out rates and the child welfare services (Open Society Foundations 2013); experiences of humiliation in the interaction with Norwegian authorities (Fangen 2006); and, the dual identities of young Norwegian-Somalis (Fangen 2007). The contributions focusing on health aspects of the Norwegian-Somali group include studies on the practice of genital mutilation (Ziyada *et al*. 2020); cultural differences in perinatal care (Glavin and Sæteren 2016) and gender roles and mental illness (Næss 2019).

One recent particularly relevant Norwegian study discusses the role of trust in the health care integration of Somali immigrants in Norway. Using qualitative data from a birth clinic and everyday consultancy, Næss (2019) describes the relationship between Somalis and Norwegian heath care workers as ‘characterized by a pervasive, mutual unfamiliarity’ (Næss 2019: 1). Næss (2019) further notes the value of persons of Somali descent with cross-cultural health competency as bridge-builders possessing what he labels a cultural health capital.

### A Comprehensive Welfare State

Norway has a comprehensive welfare state and a well-established universal healthcare system. The Norwegian health services, including primary doctors and hospitals in Oslo, were mobilized at an early stage of the pandemic. Formal access to healthcare should not be a factor in the Somali reaction to infection prevention information. This article does not discuss how different subgroups within the Somali community in Oslo vary in their propensity to contact public health services.

### The COVID-19 Crisis in Norway

On 12 March 2020, the Norwegian Government announced a national lock down, a strategy seeking to reduce the spread of the COVID virus (https://www.regjeringen.no/no/aktuelt/nye-tiltak/id2693327/). Schools and kindergartens were closed, travel restrictions were implemented and quarantine measures were introduced. A key component of the message from the government was a list of advisory points regarding social distancing and personal hygiene (https://www.fhi.no/contentassets/37298bcaf6724377b68018cfd7db4309/vedlegg/english_generell-informasjon-korona.pdf). At the podium of the press conference that day were among others the Prime Minister, the Minister of Health and Care Services and the head of the NIPH. The event was a major televised media event (Ytreberg 2017) that echoed on all media platforms, including social media, in the hours and days that followed. The government representatives gave daily press conferences on the development of the spread of the virus, making sure to repeat the key points of advice with regard to social distancing and hygiene. This information was repeated on the web pages of national institutions (the government, ministries, directorates, NIPH, HelseNorge (National Contact Point for Health Care (https://www.helsenorge.no/))) and also at the municipal and city district levels. During March, the information was translated into a host of languages. By the end of the month, the NIPH pages included translations of the key individual measures in more than 40 languages (https://www.fhi.no/nettpub/coronavirus/infomateriell/generell-informasjon-koronavirus-pa-flere-sprak/). The written information was supplemented by videos published on YouTube in 15 languages, including Somali (https://www.youtube.com/playlist?list=PLuArDsXNEjNLV95bgBpZfijDbZ35buTZy). Parallel to these information efforts, the number of hospitalizations continued to increase, reaching peak numbers on the very last day of March.

**Figure 1:**
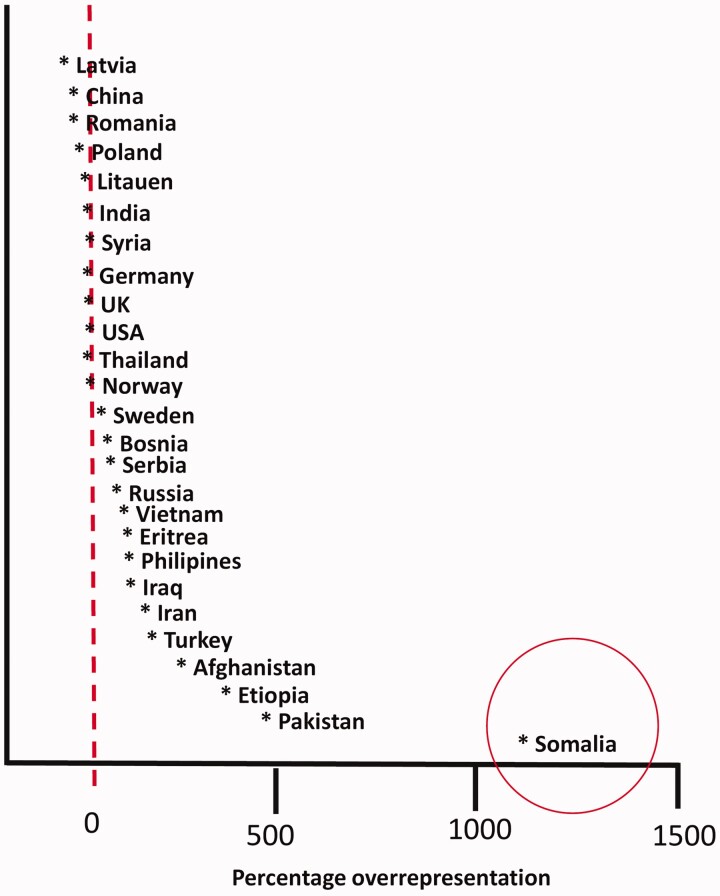
Confirmed Covid-19 infection among immigrant groups in Norway compared to majority population. March–June 2020. Percentage overrepresentation (Source: Norwegian Directorate of Immigration 2020).

The overrepresentation of Somali immigrants among those hospitalized was reported in the media on April 1. This was at the very height of the number of hospitalizations and still at a time of high uncertainty on how the spread of the virus would develop. For politicians, bureaucrats and health personnel at the national, municipal and city district levels, concern over this overrepresentation came on top of an already unprecedented level of urgency. As seen in Figure 1, there were major differences in infection.

While the matter was discussed at the city level in Oslo, immediate action was taken at the city district level. Realizing that the methods implemented up to that point, mostly information in major Norwegian printed media and on Norwegian TV and radio, had apparently failed to reach all parts of the Somali community, city district employees looked for a new information strategy. They started out by setting up a meeting in the local mosque with Somali representatives.

## Concepts of Communication

The goal of the Norwegian Government, at both the national and local levels, was to reach all groups of the population with information on how to avoid contracting the coronavirus. One way to conceptualize these efforts is to liken them to information campaigns. Theory on such campaigns typically distinguishes between inputs, outputs and outcomes (Macnamara 2014).

Inputs are the messages and the presentation of the information and the choice of channels or platforms and strategies to reach the intended audience. Outputs are observable results, such as the number of messages sent, the number of people who received them and secondary media coverage. The outcomes of the campaign are concerned with the number of people who changed their attitudes or behaviour because they were exposed to the campaign (Brekke and Beyer 2019).

The interviews in this article were held at the height of the information campaign and therefore provide more information on the input and output side of the information efforts than on the actual outcomes—the effects, such as the changed attitudes and behaviour of the Somali population with regard to infection prevention. However, the informants made assumptions about the effectiveness of their efforts. Therefore, key concepts on the effectiveness of information campaigns will also be presented.

Basic campaign theory further distinguishes between the following four elements of the communication process: the sender, the message, the channel and the receiver (Voltmer and Römmele 2002). In other words, communication efforts should be understood as a function of who, what, how and to whom it is communicated.

In the case of attempting to transmit the COVID-19 information to the Somali population, all of these elements were in contention. This cross-cultural communication setting places idiosyncratic demands on the role of the sender, the formatting of the message, the choice of channels and the in-depth understanding of the target group. The latter includes understanding their preconceptions about the senders and channels as well as their knowledge of the topic at hand. It follows that campaigns need to be context-specific, taking into consideration the situation of its intended audience, tailoring the messages to this target group (both overt features such as text and symbols as well as inert—the meaning and intention of the message) while using platforms and channels relevant to this group.

The role of communication ambassadors resembles that of opinion leaders, described in Lazardsfeldt *et al.*’s (2020) two-step communication model. This theory suggests that interpersonal interaction has a far stronger effect on shaping public opinion than do mass media outlets and that media content first reaches ‘opinion leaders,’ people who are active media users and who collect, interpret and diffuse the meaning of media messages to less-active media consumers (https://www.britannica.com/topic/two-step-flow-model-of-communication).

Communication theory further emphasizes the distinction between one-way (transmission) communication and two-way (interactive) communication (Voltmer and Römmele 2002). If regarded as a continuum, the extremes would be a pure-persuasion, no-feedback model at one end and a mutual, ‘sharing-of-meaning’ with no distinction between sender and receiver at the other. In-between we would find all variations of interaction.

Over the past two decades, the literature on intercultural communication in health-care has developed along two strands: One focusing on intercultural communication competence and the other focusing on intergroup cultural communication (Kreps and Kunimoto 1994). While the first concentrates on individualized cultural skills, often within health professions (Halbert *et al*. 2006), the latter looks for the dynamics that underpin the understandings and communication between groups (Watson 2008).

Scholars studying government campaigns have pointed out three key prerequisites for such campaigns to be effective in changing attitudes and behaviours (Matthes 2005).

Firstly, the target population, in our case the Somali population in Oslo, has to experience a need for the information. The concept used in the general theory of media influence and agenda setting is the need for ‘orientation’ (Weaver 1980). For a message to be effective, the recipients have to realize that they have a need for orientation. This need again depends on two conditions—is the information relevant and does it fill a knowledge gap? If the target group perceives the information as relevant and as covering a topic where there is uncertainty, this will contribute to its effectiveness.

Secondly, the timing of the information is key. Does the target population experience the information as relevant at the time when they receive it? At which point in time did the hard-to-reach groups within the Somali population in Oslo perceive the information as relevant?

Thirdly, do the recipients of the information see themselves as the target of the information campaigns? If not, they may ignore the information as not intended for them.

Finally, studies of campaigns and mass communication point to the role of other information sources (Klapper 1960). In our case, the informants discussed the role of information from Somali news sources, social networks in Somalia and networks in other third countries.

## Methodology and Data

The case of the Somali ambassador campaign in the Gamle Oslo city district was chosen in the direct aftermath of the news of Somali overrepresentation. This district is the area with the highest density of people of Somali descent in Norway and therefore stood out as the key area to study the challenges involved in communicating infection prevention. Through contacts in the Oslo city council, doors were opened to the city district administrative staff. These, in turn, provided contact information to the ambassadors within the Somali community.

Informants were contacted through the city district staff engaged in the information campaign. The following two groups of informants were identified: five civil servants in the city administration and in the city district of Gamle Oslo and five of the information ambassadors of Somali descent. The city district administrators selected the ambassadors based on information about and gained from the Somali community itself. The process and criteria are described below. All key actors in the district of Gamle Oslo were interviewed as were five out of six of the information ambassadors.

Semi-structured open interviews were conducted with the 10 informants. This method allows for following the specific competencies and interests of the individual informant, while at the same time securing cross-interview comparison. This format also made it possible to include topics that surfaced during the first few interviews as part of the later interviews, such as different types of trust and the role of health experts as ambassadors.

The interviews were conducted during the Easter holiday week. Interestingly, both groups of informants were willing to set up appointments during this time. Many of the civil servants were working close to around the clock and had been doing so since the start of the crisis. The interviews were seen as part of the ‘dugnad’—the community efforts to stop the spread of the virus. The information ambassadors were also highly motivated and went out of their way to schedule interviews during the holiday week. All informants experienced the situation with the virus continuing to spread within the Somali community as grave and welcomed this opportunity to share their experiences.

As a consequence of the pandemic, the interviews were held by video communication (Skype and others) or telephone. Since the interviews were done either with civil servants outside of working hours or with the ambassadors outside of their regular working roles, and as a result of being confined to their home offices/home settings, the video interviews highlighted the extraordinary circumstances of the situation.

Written notes from the interviews were analysed by first coding sections with overlapping content from the different informants, next by comparing these coded sections looking for patterns linked to the theories on campaign effects.

The ambassador campaign was limited to the Somali community and to the city District of Old Oslo (DOO) during the first wave of the pandemic. Most of the ambassadors were residents of this part of Oslo, while two lived elsewhere in the city. After the first wave, similar communication models have been implemented adopted elsewhere in the city and in other places in Norway, often targeting more than one immigrant group (https://www.dagbladet.no/nyheter/smittes-rundt-middagsbordet/73405520).

### Anonymity

With a limited number of interviews located within a specific geographical area, securing the anonymity of the informants is a key concern. At the same time, the local context and the characteristics of the city district provide important information for the interpretation of the data and for securing further relevance of the findings for information efforts elsewhere. As a result, the names and other identifying characteristics of the informants have been changed in this article, while the name of the city district has not.

### Ethical Considerations

The communication ambassadors described in the interviews a reluctance to take part in the special efforts to reach the Somali population in Oslo. The reason was that this group is often highlighted as the nationality with the lowest labour market participation and low scores on living condition parameters. The belief was that the campaign might contribute to the group’s stigma (Goffman 2019; Logie and Turan 2020). Such considerations were also relevant for this study. Could the research in itself contribute to further stigmatizing the immigrant group and therefore inflict harm? In this case, the potential benefits of the study—learning about how to succeed in communicating vital information to groups that are hard to reach—are seen as trumping such potential negative consequences.

## Results

In the interviews, the city district employees stressed the importance of the strategic choices they had made at the start of the project. Together with the communication ambassadors, these focused on securing the legitimacy of the senders, identifying effective formats and channels, knowing the target group and securing the intended outcomes.

### Deciding on a Communication Strategy

At the end of February 2020, 2 weeks before the national lock-down, the staff in the city’s District of Old Oslo (DOO) established a preparedness committee. The goal was to secure information to the inhabitants and to be in a position to handle emerging outbreaks. The committee reviewed the information directed at immigrant communities that was produced by the NIPH and the central administration in the municipality of Oslo during the first weeks of March. They found the information lacking.

*The NIPH and the Municipality of Oslo were providing some information in other languages during the first weeks of March. However, the information was a mix of translated written text and symbols. We immediately saw that this would not suffice. We very much doubted that the information would reach the target audience. (Employee DOO)*

A list of measures was implemented simultaneously, including direct contact with immigrant youths, visits to immigrant stores and posters being put up throughout the district. Parallel to these efforts, the city district saw the need for a separate communication strategy to reach the Somali population, an immigrant group, parts of which were considered to be hard to reach.

*A number of information measures were running parallel at that time. However, despite these and all the resources that went into them, we did still not manage to reach certain groups of the immigrant population. There is no doubt that many had heard of the national guidelines for preventing infection, but still they did not follow them. (Employee DOO)*

By the end of March, reports were coming from Sweden of persons of Somali descent being overrepresented in the statistics on hospitalizations due to COVID-19. This was quickly followed in early April by reports from the NIPH mirroring the findings from Sweden. A few days earlier, the city district had received backing from the leader of the city council for the new information strategy, labelled ‘information ambassadors.’ Here, the idea was to secure information to the Somali community by applying a bottom-up instead of a top-down approach and using members of the community as volunteer expert communicators.

The first step in the strategy was to organize a meeting with representatives of different parts of the Somali community at a local mosque. The district employees pointed out in the interviews that they invited themselves to the meeting, thereby stressing the equal footing of the two parties. However, the representatives from the Somali community were already engaged and saw mutual interest in a prompt effort to disseminate information about infection prevention.

At the meeting, the main elements of the communication strategy were established, including the role of the senders of information, the format of the message, what channels to use and the target group. Three weeks later, during our interviews, the informants commented on these same elements, adding their thoughts on the outputs, relevance, timing and outcomes.

### The Senders—Ambassadors Bridging the Majority and Minority Gap

At the Mosque meeting, it was established that to secure the credibility of the information, it had to come from a person with Somali background. All informants agreed that the persons chosen needed to be trusted by the hard-to-reach groups within the Somali community. According to the informants, this included having a solid footing in both the minority and the majority communities and having established authority. If those fronting the communication would additionally be educated health personnel, that would be an advantage. Following this tall order, six ambassadors were recruited during a 2-day stretch using existing contacts within the Somali community in the public health services and personal contacts.

The informants pointed out that the existing information effort had not been effective because the senders did not have credibility. Two cases were mentioned by all of the informants of Somali descent—a video made by the Oslo Police (https://www.facebook.com/NorSomNews/videos/211671433385525/?t=73) targeting the Somali community and a video made by the Norwegian Broadcasting Cooperation (NRK). Both prompted strong reactions among the community representatives.

*When the police published the video, where they told the Somalis in Oslo to stay at home, that was a catastrophe. They criminalized the Somalis, again! Why would the police communicate basic rules for how to avoid the spread of the virus? Shouldn’t health experts be doing that job? (Ambassador)*

The experience of being an already-stigmatized group created a sensitivity toward the choice of using the local police to inform this particular immigrant community.

*Having the police target one particular ethnic group with this information is distasteful and sad. Young people with a Somali background experience ethnic profiling by the police, and such a video makes things worse. This is not our police. They should have contacted resources within the community instead of targeting us like that. (Ambassador)*

The police officer used in the video did not have a Somali background, nor was he a health expert. Given the tensions between the Somali community and the police, a police officer was not regarded as a credible source. One informant of Somali descent disagreed with the condemnation of the police video. He held that ‘The police video made people realize how important this was. They may have opened doors for further communication, which could be helpful to us’ (Ambassador).

In the NRK incident, a journalist of Somali descent presented basic anti-dissemination advice in line with the NIPH guidelines (https://www.nrk.no/video/sagal-informerer-om-Covid-paa-somali_8d0fac02-e7b8-4e9b-bf12-d609400e879c). The video was in Somali with Norwegian subtitles. According to the informants, however, the journalist was not a health expert. In addition, she was perceived as not speaking Somali sufficiently well. This created reactions within the Norwegian and extended Somali communities:

*It was so embarrassing to watch. I am sure the journalist was good at her work, but her Somali was so bad that it was difficult to understand what she was saying. I had to read the Norwegian sub-titles to get her message. (Ambassador)*

The informants lauded the efforts made by the state-run TV channel to reach out quickly to the Somali community but pointed to the lack of quality checking and their misperception of the target group.

*The intention was good. Informing in Somali. If they wanted to reach the first-generation immigrants, however, they failed. The poor journalist, who had grown up in Norway, was later ridiculed within Somali networks on social media. (Ambassador)*

Several informants pointed to the crucial role of the senders having excellent language skills. Since the group deemed hardest to reach were first generation and knew little or no Norwegian, the ambassadors had to know Somali as well as the first-generation immigrants. The informants with Somali background also remarked that Somali is primarily an oral language with only a more recent written tradition. They saw Somali subtitles as less effective, putting even more weight on presenters being well versed in spoken Somali.

The informants stressed the need for the ambassadors to have authority and competence. In addition to three medical doctors, one engineer and two teachers were recruited. These were all active participants, often volunteering within the local community. They were, however, not representatives of organizations within the Somali community but rather standalone individuals with a certain level of recognition and high standing. This secured the neutrality of the ambassadors; they had no potentially divisive ties to parts of the community.

The ambassadors saw the competence of those who fronted the campaign to be crucial.

*You cannot have a plumber explaining how COVID-19 affects the human body. The message has to come from trained health experts. They have to know what they are talking about, and people have to know that they are experts. (Ambassador)*

Most of the ambassadors had already taken private initiative in helping their community when they were recruited. As one of the medical doctors put it:

*I contacted the NIPH days before the city district called. I had seen the numbers from Sweden and heard that Swedish-Somali doctors were worried. I asked NIPH for numbers on Norway and told them that I was a doctor with a Somali background. They suggested a communication model similar to the ambassadors: link-employees. (Ambassador)*

Some of those contacted refused to take on the role as ambassadors. According to the employees from the district administration, this was due to the perception of a rift between the majority and minority populations.

*Some did not want to take part. They were a bit reluctant to end up as spokespersons for the authorities. There could be several reasons for this, of course, but some experience that the media and the authorities stigmatize the Somalis. The do not want to be seen as representing these same authorities. (Employee DOO)*

Once the ambassadors had been recruited, they started producing information videos in cooperation with the staff of the district administration. Together, they decided to spread the videos only within the Somali community and not post them on, for example, the webpages belonging to the municipality. This strategy had consequences in regard to who was regarded as the senders of the message. They featured experts from the community but were distributed from one Somali to the next.

### Message and Channels

Together, the employees from the district and the ambassadors decided on the content and the format of the message in the videos during the last week of March. The basis of the message was a list of measures published by the NIPH in the previous weeks to prevent the spread of the virus. These included advice on what to do to prevent dissemination as well as what to do if infected. The list was then modified according to the questions and comments from the Somali community members discussed at the mosque meeting, such as the difference between quarantine and isolation. The advice from the ambassadors included the need to use simple language and include concrete instructions.

*We had to simplify the message. This was particularly important in order to reach the older population. Instead of saying droplet infection, one should be concrete and talk about spit coming out of your mouth or spit on door handles coming into your mouth or nose. (Ambassador)*

Pointing to Somali being primarily a spoken language, the message in the videos was delivered in Somali with Norwegian subtitles, thereby reaching out to the second generation.

The campaign team—the ambassadors and the city employees—decided not to use symbols as part of the communication. Symbols had been used as part of the existing dissemination efforts by the municipality of Oslo and were featured on posters around the city. The campaign team pointed out that there was a risk that some members of the Somali community would interpret the symbols differently than intended. The example they mentioned involved Somali mothers being asked to rate a particular course held on the topic of children and nutrition by ticking pictures of smiling, straight or sour faces. Despite being happy with the course, they had all ticked the straight face. When asked, the mothers pointed to the fact that this was a serious topic and not a laughing matter.

The videos were distributed through private channels only, and the employees in the city district had to defend this decision when the central administration of the municipality and other city districts wanted to share the videos on their web platforms. They had to stand by the principle that the videos should only be distributed from one person of Somali descent to another. They did, however, offer a link to the video to these colleagues on the premise that they would give it only to their contacts within the Somali community. ‘This is how news is normally distributed within the community,’ according to one of the ambassadors. According to the informants, 2 days after being published on YouTube, 20,000 persons had watched the first video.

Parallel to the publishing of the video, the ambassadors pushed the message in other channels, such as in special programs on the most popular Somali-only local radio-station, through direct contact within personal networks and face-to-face fielding.

At the height of these efforts, during the first week of April, news spread within the Somali community that one of their community members had died of COVID-19. According to the city district employees, this spread fear within the community and may have resulted in increased adherence to the infection prevention advice.

*The news of his death immediately spread throughout the community. He was an active member of the local mosque and known as being a careful man. This led many to think: ‘If this could happen to him, it could happen to everyone.’ (Employee DOO)*

### The Target Group

The informants pointed out that the group of individuals of Somali descent living in Oslo is diverse.

*The majority society often see the Somali community as one group. But, this is a very diverse group, encompassing medical doctors and illiterates. What is clear is that we do not understand the diversity involving family and clan structures. (Employee DOO)*

Informants of Norwegian descent highlighted this lack of knowledge of the social dynamics within parts of the Somali community.

*We know little of where they meet and how they spend their days. We have been told that there are groups of single men who eat all their meals outside their homes. Now, those restaurants are closed, this is a real problem. (Employee DOO)*

How did the hard-to-reach parts of the Somali community get their news and information about the Norwegian society? According to the informants, whereas many within the community would have the Norwegian main stream print media, TV and radio as well as Norwegian webpages and social media as their information sources, this more isolated group did not follow Norwegian media, or did so only to a very limited degree.

Instead, they rely on social media, networks, Somali news sources and the dedicated Norwegian-Somali radio station.

*Young persons will typically use other platforms than the older generation; have a different level of contact with family abroad and in Somalia, etc. However, they all use Facebook. WhatsApp is a very important platform according to our contacts. They also follow the NORSOM radio. Finally, they follow international news sources, such as Arabic TV-stations. (Employee DOO)*

The ambassadors saw the challenge with parts of the community relying too much on Somali news sources:

*We have a lot of Somali TV-channels, but they focus on conditions in Somalia. There is no TV for Somalis in Norway. Except for the one radio-channel, NORSOM, one is left with social media and the web, where it can be difficult to know what is correct information. (Ambassador)*

According to the ambassadors, many in the older generation know only rudimentary Norwegian and do not know enough Norwegian to follow the news.

*They can go to the store, etc., but in order to understand and interpret the main-stream news, you need a completely different level of understanding. You may even need to be an academic in order to understand what is being said at the press conferences. (Ambassador)*

The informants from the city district administration confirmed the impression that much of the information was too advanced and needed to be simplified and made concrete.

*We have to remember that much of the health information is made for the (white) upper middle class. We often talk in a way that makes it impossible to follow. We made an effort to simplify the language, but there is room for improvement. (Employee DOO)*

The informants mentioned that the strong care culture within the Somali community may have contributed to the challenges of reaching the target group. Both the employees and the ambassadors pointed to a strong culture of voluntary care assistance within the community. This made reaching them all the more important.

*There is a lot of voluntary work within the Somali community, often organized around resourceful women, who organize the help. It must have been difficult for many Somalis to abstain from helping those who were sick, thereby risking further spreading the virus. (Employee DOO)*

The helpers within this separate informal care system needed to abstain from contacting those in need to adhere to the anti-dissemination guidelines.

### Outcomes

Based on the interviews alone, it is not possible to estimate the actual impact of the video campaign on changing the attitudes and behaviours of the target group.

*It is difficult to estimate the effects of this particular campaign, but the attitude toward the infection has changed. We see a big change now. The police report fewer Somalis in the streets. People, who did not take it seriously do now. (Ambassador)*

The informants did, however, point to the spread of videos and had thoughts about changes related to the criteria of the need for orientation, relevance, uncertainty and timing.

The videos went viral within the Somali networks. Twenty-four hours after the first video was published through the ambassadors’ Somali social media networks, the YouTube-based video had had 20,000 registered views. In total, the campaign team produced four videos, featuring two of the medical doctors who had volunteered as ambassadors.

The death of the community member highlighted a shift in the target group’s need for orientation according to our informants. The hard-to-reach subgroups within the Somali community had been slower to realize the seriousness of the virus situation than the rest of the population. The news that Somalis were overrepresented in the hospitalization statistics, and individual stories from Sweden, increased the individuals’ need for orientation. The information was obviously relevant, and one must expect that it was experienced as such by the subgroup once the need for orientation had opened the door for the input. The city district’s campaign also addressed an area marked by uncertainty. There was still widespread uncertainty about the virus and the best way to protect oneself and family and friends at that point in time, both in the minority and majority populations. The final element regarding the potential outcome, the timing, also pointed toward expected effectiveness. The campaign produced outputs quickly and could speak to the immediate situation in the local area. In sum, the factors often connected with effective campaigns were present.

## Discussion

The ‘ambassador information campaign’ in Oslo during the height of the Corona outbreak in the spring of 2020 revealed both the challenges involved in reaching largely isolated subgroups within an immigrant community with vital information and important learning points on how to succeed in doing so. In addition, the interviews raised questions about the consequences of unequal access to health information within a city’s population and highlighted the distance between a well-informed in-group and a segregated and largely uninformed out-group.

### Challenging the Traditional Communication Strategies

Additionally, the interviews demonstrated that the traditional way of communicating about health and infection prevention was not effective in reaching the hard-to-reach groups within the Somali community in Oslo at the start of the coronavirus outbreak in the spring of 2020. There was an urgent need to rethink all aspects of the communication strategy, including the role of the senders, the messaging, formats, outputs and the understanding of the target group.

As to the role of the senders, initially, the message senders were Norwegian authorities at the national and local levels. Their outputs (messaging) were in Norwegian and often perceived as being too complicated, especially being given via in-group formats such as the majority’s media (TV, radio, newspapers) and web pages of public institutions. Some texts were quickly translated into Somali. However, as the interviews show, few within the community understand and yet fewer use, written Somali. The authorities were unaware of possible challenges with the cross-cultural interpretation of symbols and illustrations. In addition to this, the authorities did not appear to have the necessary understanding of the target group and in particular of hard-to-reach subgroups within the Somali community.

The next wave of information directed at the Somali community included videos with people speaking in Somali. These outputs, initiated by the NIPH and the NRK, fell short due to the senders not having sufficient competence and standing within the community or not knowing the language well enough. As a result, despite choosing the right format—videos with spoken Somali—these senders lacked the necessary legitimacy within the Somali community, and the persons delivering the message did not have relevant competency. In addition, in this wave, the NIPH and NRK used traditional channels (TV and institutional web pages) rarely visited by the hard to reach within the community.

Zooming back out, at the start of the pandemic, the Municipality of Oslo had limited knowledge about how to reach the least-integrated groups within the Somali community. One could say that their cultural intergroup communication competency (Watson 2008), was limited. The serious nature of the COVID situation highlighted the need for such knowledge and relevant strategies.

The interviews indicated that groups within the Somali community live outside of the public stream of information and may be unaware of their rights and duties as part of the larger majority society. The crisis revealed a communication gap that, in turn, pointed to groups living in deep segregation from the mainstream societal life.

Zooming back in, the eventual ambassador campaign *did* meet all of these requirements by selecting in-group experts with language skills as senders, crafting messaging based on the questions the target group wanted answered, using relevant channels and having a profound knowledge of the target group. The drawback of the model, however, was that it entailed social risks for the individual ambassadors.

The reluctance of some of the communication ambassadors to join the campaign points to two challenges involved in serving as a bridge between the majority and the minority Somali community. They believed that by joining, they may be contributing to the negative stigma of the Somali community. They also realized that by being part of a campaign initiated by public institutions, they could risk personal costs by speaking on behalf of the majority population ‘down’ to the Somali community. These risks made some candidates reject the campaign, while others joined, citing the urgency of the situation.

The ambassadors volunteered or received minimal compensation. The voluntary aspect contributed to being able to both feel and be perceived as independent communicators, operating in-between the original senders, being the local government and the Somali population. However, this format as non-employees of the municipality also challenged the sustainability of the ambassador model. When they were asked to prolong their services and make more videos at a later and less critical stage of the spread of the virus, they were less eager to take part.

The ambassadors were knowledgeable of both the language and the social and cultural situation of the target population of the campaign. The ambassadors were persons with a high societal standing; being experts gave their voices credibility. Those with professional health background benefitted from their cross-cultural health expertise. These informant ambassadors stressed the role of communicating from one person with a Somali background to another. By speaking horizontally, meaning ‘with’ and not ‘to’ the group, they avoided possible rejection by not talking down, vertically, to the community. The channels used, spreading the messages and videos through personal and community networks only, contributed to the campaign’s success.

The interviews revealed that traditional means of top-down, majority-to-minority communication did not suffice in reaching hard-to-reach groups within the Somali community in Oslo. The communication challenges also pointed to the risk of not being able to fulfill a key principle in the Norwegian welfare state of equal access to health services, a principle that presupposes (equal) access to health information.

The data indicate that the municipality made strategic use of the ambassadors as opinion leaders (Lazardsfeldt *et al*. 2020). While the Somali ambassadors were well connected within the majority and mainstream public and media sphere, other members of the Somali community were not and thus looked to them as intermediaries, as bridging communicators and translators of information and knowledge.

The independent role of the ambassadors helped secure the horizontal trust within the community. They did not represent a voluntary organization nor were they regular employees of the local government. The ambassadors thereby avoided questions of mistrust and hidden motives said to be frequent within the Somali community, such as *Why is she saying this?*, *Who does she represent?* and *Who is paying her to say this?* The interviews indicate that despite showing high levels of institutional trust and interpersonal trust in surveys, there may actually be lower levels of trust within the Somali community. The ambassadors’ independence, competency and standing seemed to overcome these challenges, combining vertical and horizontal intra-group trust. The campaign thereby displayed the key role trust plays as a foundation for communication.

The ambassador campaign simultaneously highlighted the communicative distance between the majority population and its institutions, on one hand, and pockets of hard-to-reach members of the Somali community on the other. These members lead lives communicatively segregated from the mainstream society. This COVID-19 crisis revealed the social and communicative distance between these two parties and its consequences.

Returning to the research question, the Somalis in the Oslo case provide important information on what is needed to communicate effectively with hard-to-reach immigrants groups. The perceived legitimacy of the senders is crucial. In the Oslo case, in-group experts with adequate language skills secured the legitimacy of the messages. The messages were formulated based on the questions the target group wanted answered and were distributed through community networks. The campaign was launched in the wake of shocking news on overrepresentation, positioned between both what was reported as ‘need-to-know’ within the community and relevant timing. The relevance of these experiences for other immigrant groups in other social, historical and geographical contexts remains to be seen.

The experiences with communicating anti-infection information in Oslo are, however, not unique. Practitioners elsewhere in the world report similar situations and solutions (https://www.mprnews.org/story/2020/05/22/st-cloud-effort-aims-to-share-covid19-info-help-in-somali-community). In Oslo, as in other places, local governments and ethnic communities have applied multiple measures simultaneously. Systematic evaluation and further research are needed to fully understand the effect and outcomes of individual campaigns, such as the ambassador initiative in Oslo. As of spring 2021, amidst a third wave of COVID-19 infections across Europe, it is clear that there will be a constant need for updating anti-infection information and its mode of delivery. Further research is needed in regard to systemizing the early lessons on how to reach segregated members of ethnic communities.

## Appendix 1. Informants.

**Table:**

	Affiliation	Gender	Role/department
1	Employee District of Old Oslo (DOO)	Female	Head of department
2	Employee District of Old Oslo	Female	Communication officer
3	Employee District of Old Oslo	Female	Integration officer
4	Employee District of Old Oslo	Female	Head of project
5	Employee city of Oslo	Female	Head of communication
6	Communication ambassador, Somali background	Male	Radio host and owner
7	Communication ambassador, Somali background	Female	Medical doctor
8	Communication ambassador, Somali background	Male	Teacher, youth contact
9	Communication ambassador, Somali background	Female	Medical doctor
10	Communication ambassador, Somali background	Female	Nurse
